# Risk of infective endocarditis in patients with mitral valve prolapse: Systematic review with meta-analysis

**DOI:** 10.1016/j.heliyon.2024.e39893

**Published:** 2024-11-04

**Authors:** Luisa Marques, Catarina de Sousa, Fausto J. Pinto, Daniel Caldeira

**Affiliations:** a Faculdade de Medicina, Universidade de Lisboa, Portugal; b Cardiology Department, Hospital Universitário de Santa Maria – ULS Santa Maria (ULSSM), CAML, Portugal; c Centro Cardiovascular da Universidade de Lisboa – CCUL (CCUL@RISE), CAML, Faculdade de Medicina, Universidade de Lisboa, Portugal; d Centro de Estudos de Medicina Baseada na Evidência (CEMBE), Faculdade de Medicina, Universidade de Lisboa, Portugal; e Laboratory of Clinical Pharmacology and Therapeutics, Faculdade de Medicina, Universidade de Lisboa, Portugal

**Keywords:** Infective endocarditis, Mitral valve prolapse, Billowing, Antibiotic, Barlow disease

## Abstract

**Aims:**

Infective endocarditis (IE) is a serious heart valvular condition. While mitral valve prolapse (MVP) has been associated with an increased risk of IE, the magnitude of this association remains poorly quantified. This systematic review aimed to better estimate the risk of developing IE among MVP patients compared with the general population.

**Methods:**

MEDLINE, Cochrane Library (CENTRAL) and Web of Science databases were searched electronically to find all the relevant cohort and case-control studies. Pooled estimates of odds ratios (ORs) and 95 % confidence intervals (CIs) were derived by random effects meta-analysis. Heterogeneity was assessed using the I2 test.

**Results:**

A total of six studies were considered eligible, and the obtained results showed that MVP patients had a higher risk of IE when compared to the general population (OR 7.83, 95 % CI 5.11, 12.02; I2 = 0 %). Posterior analysis according to the risk of bias and study design didn't show any significant variations in the direction and magnitude of the effect.

**Conclusion:**

The magnitude of increased risk of IE of 7-fold warrants further attention for patients with MVP. Further contemporary studies and prophylaxis studies should be considered.

## Highlights

• The magnitude of endocarditis risk related to mitral valve prolapse is unknown.
• Mitral valve prolapse increased more than 7-fold the risk of infective endocarditis.
• Further contemporary studies and prophylaxis studies should be considered.

## 1 Introduction

Infective endocarditis (IE) is a serious condition associated with severe complications, such as congestive heart failure, stroke, embolic events and abscess formation, which can be fatal in about 25 % of the patients [1]. While some studies report a stable incidence of 5.3–9.4 cases per 100 000 person-years, others have noted an increasing incidence in recent years [[2], [3], [4]].

IE prevalence is higher in some groups, such as patients with a prosthetic valve or prosthetic material related to valve repair, patients with a previous episode of IE and patients with a cyanotic congenital heart defect (CHD) or any CHD if repaired with a prosthetic material. In addition, any other form of native valve disease (including mitral valve prolapse) corresponds to an intermediate risk of IE [5].

Mitral Valve Prolapse (MVP) is defined as an abnormal bulging of at least one mitral valve leaflet into the left atrium during ventricular systole. MVP diagnosis is based on echocardiographic findings, which have changed over the years, as echocardiography techniques improved [6]. Prior to the year 2000, echocardiographic studies implicitly assumed that the mitral annulus was planar. This assumption led to the belief that leaflet-annular relations would be comparable in both the parasternal long-axis view and the apical four-chamber view. Superior leaflet displacement in the apical four-chamber view was accepted as a diagnostic criterion. Consequently, MVP was often diagnosed based on the apical four-chamber view, even if it was absent in the parasternal long-axis view. These criteria resulted in MVP being diagnosed in about 10 % of the general population, including preselected normal individuals, suggesting that these criteria were overly sensitive [[7], [8], [9]]. Currently, MVP is defined as a displacement of the margin of at least one mitral valve leaflet beyond the annular plane (>2 mm) during systole, with or without leaflet thickening, usually identified from the parasternal long axis view. Given this definition, the estimated prevalence of prolapse is 2.4 % [10].

Although it has been reported that MVP leads to a higher risk of IE, the magnitude of this relation is not well described. Being a common valvular disease, this association is to be expected, but the relative risk is not properly documented, nor important risk modifiers, such as the association with mitral valve regurgitation.

This systematic review aimed to better estimate the risk of developing IE among MVP patients compared with the general population.

## 2 Methods

To guide the review methods, a protocol was previously written, stating the review question (if MVP patients have a higher risk of developing IE than the general population), the inclusion and exclusion criteria, the search strategy employed and the meta-analysis plan. The protocol is available at http://osf.io/uh9p2.

### 2.1 Eligibility criteria

This review considered eligible all observational studies (cohort or case-control) reporting IE in patients with and without MVP. MVP patients were considered eligible regardless of age, gender, or disease severity.

In case-control studies, IE patients were considered as the cases and patients without IE as the controls, to then evaluate the previous existence of MVP and compare its prevalence in both groups. Regarding cohort studies, they were required to be population based, allowing to compare IE incidence in patients with and without MVP, with a minimal follow up period of 5 years.

Both IE and MVP diagnoses were accepted based on clinical medical judgement. IE diagnosis considered histopathologic, echocardiographic, and clinical findings. MVP diagnosis considered both auscultatory and echocardiographic findings. Also, the diagnosis of MVP must have always preceded the IE diagnosis.

### 2.2 Data sources

MEDLINE, Cochrane Library (CENTRAL) and Web of Science databases were searched electronically in order to find all the relevant data regarding IE risk in MVP patients. The search was conducted on the databases from inception until February 2022. No language restrictions were applied in the search strategy. Data sources and the search strategy is properly described in the supplementary data.

### 2.3 Data extraction, evaluation and synthesis

Two authors (LM and DC) independently reviewed the title and abstracts of all the citations retrieved in the database search [11]. The full-text reports of all potentially relevant studies were evaluated independently by the authors, who then selected the studies to be included in the review according to the predefined inclusion criteria. Any disagreements were resolved through discussion and consensus.

### 2.4 Risk of bias assessment

Two authors independently analysed the quality of reporting by the Newcastle–Ottawa Scale [12].

This tool assesses the risk of bias based on the comparability of study groups, selection of subjects, and assessment of exposure. The authors arbitrarily used the threshold of higher than 7 to define studies at lower risk of bias.

### 2.5 Data analysis

Statistical analysis was performed with STATA 17. Forest plots illustrating individual study results and the pooled analysis were generated using this software.

The meta-analysis was performed using the maximum restricted-likelihood random-effects method weighted to estimate pooled odds ratio (OR) with a 95 % confidence interval (CI). A random-effects model independently of the existence of statistical heterogeneity was used because studies with different designs and populations were combined. Statistical heterogeneity was assessed with the I2 test.

In addition, the results were also stratified according to study design (case control vs cohort) and according to the risk of bias obtained, in order to explore differences in the outcome estimate. The Jackknife leave-one-out sensitivity analysis was performed to evaluate the impact of a single study in the final estimates. Publication bias was assessed through visual inspection of funnel plot asymmetry with Egger's test [13]. All these results are reported in the supplementary data provided.

## 3 Results

### 3.1 Study selection

**Fig. 1:**
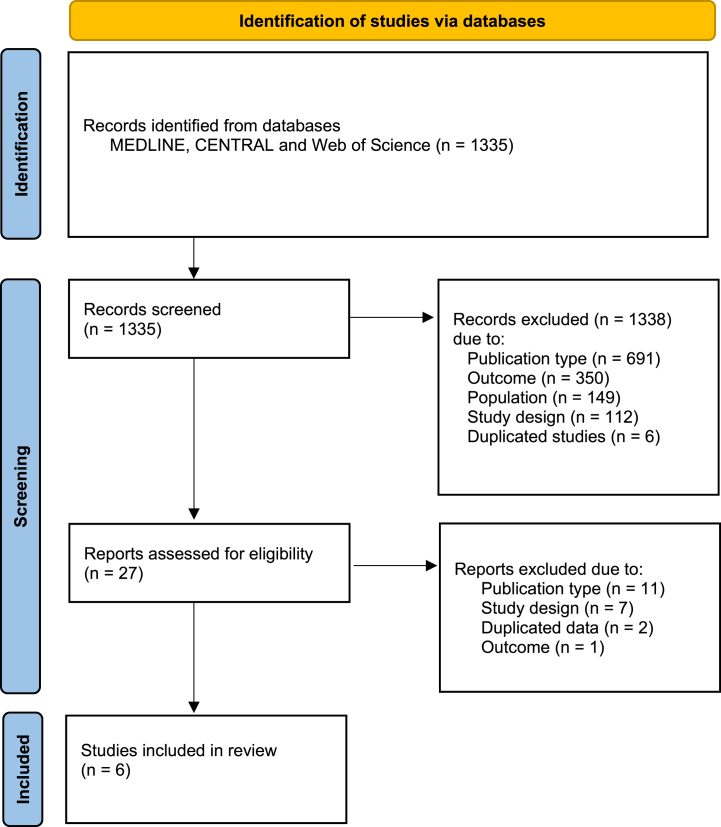
Flowchart of study selection

The literature search identified 1335 publications (Fig. 1). These publications were screened based on the titles and abstracts, which allowed to exclude 1308 of them. Full-text review led to the exclusion of an additional 21 publications, resulting in 6 studies meeting the inclusion criteria.

One case-control study (MacMahon, 1986) initially appeared to meet the inclusion criteria, but in further evaluation the cases and the controls did not correspond to the above-mentioned subjects [14]. One other contemporary case-control study (Zegri-Reiriz, 2018) could not also be included due to the absence of a control group [15].

### 3.2 Study description

Six observational studies were eligible and rendered data about the risk of infective endocarditis with patients with mitral valve prolapse. Five of these studies were case-control [[16], [17], [18], [19], [20]]; one of them was a cohort study [21]. All included studies were retrospective in nature.

The main characteristics of the eligible studies are summarized in the supplementary material. The populations studied originated from USA, Wales and France and had a wide range of ages. IE diagnosis criteria was very variable across the studies, with only one of them considering the current criteria – the modified Duke criteria [21]. Regarding MVP diagnosis, almost every study resorted to echocardiography, defining MVP as a 2 mm or more systolic displacement of at least one mitral leaflet beyond the annular plane.

To further investigate potential confounding factors in the obtained data, three possible confounding aspects were reported: the presence of another cardiac disease/lesion that could also contribute to a higher risk of IE, the existence of dental work history in the subjects evaluated and the inclusion of intravenous drug users. Some of the studies did a posterior investigation on some of these factors by excluding some of the subjects mentioned above, and reported that there still was a statistically meaningful association between MVP and IE [16,18,19].

The odds-ratio/relative risk obtained in each study and used in the meta-analysis is presented on Table 1, Table 2.

**Table 1:** Odds-ratio (OR) obtained in the case-control studies.

Author, year	Nº of cases	Nº of controls	Cases with MVP	Controls with MVP	OR (95 % CI)
Clemens, 1982	51	153	13	10	7.2 (2.1, 25.5)
Danchin, 1989	48	96	9	6	3.5 (1.1, 10.5)
Devereux, 1986	67	134	11	3	6.7 (1.96, 22.9)
Hickey, 1985	56	168	11	7	5.3 (2.0, 14.4)
Strom, 1998	273	273	52	6	19.4 (6.4, 58.4)

MVP: mitral valve prolapse; OR: Odds Ratio; CI: Confidence Interval.

**Table 2:** Estimates obtained in the cohort study.

Author, year	MVP cohort population	IE cases	IE incidence in general population	RR (95 % CI)
Katan, 2016	896	8	6.0/100 000 person-years	8.1 (3.6, 18.0)

IE: infective endocarditis; MVP: mitral valve prolapse; RR: Risk Ratio; CI: Confidence Interval.

The assessment of quality of the six observational studies was performed using the Newcastle-Ottawa Quality Assessment Scale. The full assessment is reported in the supplementary material.

Regarding case-control studies, all the selected studies matched cases and controls for age and sex. All of them retrieved the cases from a consecutive series and used the same method of ascertainment of MVP for both cases and controls. Two studies (*Clemens* et al. and *Strom* et al.) had a higher risk of bias, the first mainly due to the poor IE definition and inadequate control selection, the second due to being based on medical records only. The cohort study [43] also presented a low risk of bias.

### 3.3 Statistical analysis

**Fig. 2:**
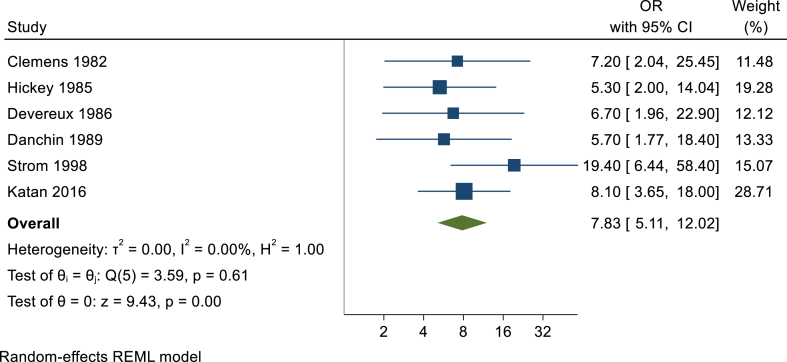
Forest plot evaluating the risk of infective endocarditis in patients with MVP.

The analysis of the six studies showed that MVP is associated with a higher risk of developing IE (OR 7.83, 95 % CI 5.11, 12.02; I2 = 0 %), as showed in Fig. 2.

The results obtained in the remaining conducted analysis (forest plots for NOS and study design subgroups, sensitivity analysis and publication bias) can be consulted in the supplementary material.

## 4 Discussion

The primary finding of this review is that MVP is associated with an approximately eight-fold increased risk of developing IE, with this estimate being consistent across the included studies. The analysis according to the risk of bias and study design were similar, without of significant variations in the direction and magnitude of the effect.

The eight-fold higher risk of IE in MVP patients has important implications for both primary and secondary prevention strategies. While this finding alone may not be sufficient to justify broad antibiotic prophylaxis for all MVP patients, it emphasizes the need for careful consideration in high-risk subgroups, such as those with concomitant mitral regurgitation. More importantly, this knowledge can inform clinical practice by promoting heightened awareness among healthcare providers, potentially leading to earlier diagnosis and treatment of IE in MVP patients. Future research should focus on developing targeted screening and management strategies for this high-risk population, balancing the potential benefits of early intervention against the risks and costs associated with overdiagnosis and overtreatment.

MVP is currently considered a condition that puts their carriers into the intermediate-risk group for IE [22]. However, this definition was based solely on case series, which don't provide the most accurate data [[23], [24], [25], [26]]. In addition to our findings, an analysis of a contemporary registry of patients with IE showed that MVP patients had a higher likelihood of having endocarditis with an odontological source compared with other low/intermediate risk patients and also compared with high-risk patients. Both together suggest that MVP classification should be revisited [15].

This risk classification is of outmost importance, since MVP does not indicate for periprocedural antibiotic prophylaxis (AP) in odontological procedures - it is currently reserved for high-risk conditions in both the European and the American guidelines [5,27]. It is important to note that these guidelines are not based on RCTs, but in observational data, given the difficulty of conducting RCTs in this context. Due to the reduced incidence of IE (even in patients at higher risk), the sample size and follow-up required for RCTs would be very large.

ESC recommendations were recently restricted to patients with a prosthetic valve or prosthetic material related to valve repair, patients with a previous episode of IE and patients with a cyanotic congenital heart defect or any congenital heart defect if repaired with a prosthetic material, up to 6 months after the procedure or lifelong if residual shunt or valvular regurgitation [5]. Posterior reviews consider this restriction to be appropriate, according to AP efficacy in preventing IE and subsequent complications [28]. However, some studies have been reporting a rise in IE incidence in the last few years – a causal relation was not yet established, but this increase can be related to this recent AP restriction [3].

The opinions on AP are increasingly divergent; currently in the United Kingdom, AP is not recommended [29]. This decision was largely due to two reasons: Firstly, the risk of anaphylactic reactions, which could represent a high death risk and subsequently made AP less cost-effective [30]. However, other studies on the subject reported the contrary, with AP having no deaths associated with antibiotic administration [31]. The second reason was that the risk of bacteraemia associated with everyday activities, such as toothbrushing, exceeded the risk of dental extractions and other procedures [32]. Even with these findings, current reviews seem to find that AP guidelines are preventing IE after invasive dental procedures and should be followed accordingly [33].

On the other side of the spectrum, other countries (such as Brazil) still have not adopted the current guidelines, maintaining the less restricted recommendations and advising for AP in MVP and other valve diseases [34].

Furthermore, even though MVP is not considered an high risk condition for IE, some surveys have shown that physicians tend to view native valve disease carriers as high risk patients, and subsequently employ AP on these patients, thinking that they fall under the recommendations spectrum [35,36]. The opposite is also happening, with some studies reporting not only a decrease in patients with low-risk lesions being advised to practice AP, but also in patients with high-risk lesions, probably due to confusion arising from these guideline changes and divergencies [37,38].

AP guidelines consider not only the risk of acquisition of IE, but also whether the underlying cardiac condition also affects the outcome of IE. In several studies, MVP seemed to be the valvular defect that was most frequently associated with IE occurrence [20,39]. Also, IE can also lead to valvular destruction, worsening the pre-existing prolapse. In this context the existence of mitral regurgitation which is thought to be a risk enhancer might also be the consequence of IE [16,17,19,21,40].

### 4.1 Limitations

Nevertheless, this review still has some limitations. Even though the stratified analysis based on the risk of bias (Fig. 2) had almost no effect on the results obtained, most of the studies eligible for this review were written between 1980 and 2000 and considered diagnosis criteria that are not currently accepted. This is more preponderant when considering IE diagnosis, given that the modified Duke criteria were only proposed in 2000 [41]. MVP diagnosis criteria, on the other hand, has been similar since mid-1980s, although the 2 mm displacement criteria only became commonly used in the 1990s [10].

Also, the studies considered in this review did not took into account several other factors that could contribute to IE development, as is reported on Table 1. Subjects could present with some other pre-existing cardiac disease or lesion that could also lead to a higher risk of IE. The same can be said for intravenous drug users, who were not always excluded and are established as a higher-risk population due to the association of endothelial damage, high injected bacterial loads and immune suppression [42]. Dental work also was not approached in these studies, and it has a preponderant role in IE pathophysiology, being the most common form of instrumentation for which AP may be needed.

Also the retrospective nature of the included studies and the predominance of case-control and cohort designs introduce potential biases. Selection bias may be present in case-control studies, while recall bias could affect the reporting of MVP diagnosis or IE events. The effect sizes observed should be interpreted cautiously, considering these potential biases and the heterogeneity in diagnostic criteria across studies. Despite these limitations, our meta-analysis provides the best available evidence on the association between MVP and IE risk.

Although our findings seem to be of relevance, given these limitations we could benefit of more recent studies, using the current diagnostic guidelines and reducing the previously identified confounding risk of bias.

## 5 Conclusion

In conclusion, this systematic review and meta-analysis demonstrates a substantial association between MVP and an increased risk of IE. This result does not have the robustness to change AP recommendations but suggest this issue merits further research. Due to the limitations in the included data, contemporary studies should be conducted in the future, using the current diagnosis guidelines. In addition, further studies should be conducted on IE risk on MVP patients with mitral regurgitation.

## CRediT authorship contribution statement

**Luisa Marques:** Writing – original draft, Methodology, Investigation, Data curation. **Catarina de Sousa:** Writing – review & editing, Investigation. **Fausto J. Pinto:** Writing – review & editing, Validation, Supervision. **Daniel Caldeira:** Writing – review & editing, Supervision, Methodology, Investigation, Formal analysis, Conceptualization.

## Funding

This research received no specific grant from any funding agency, commercial or not-for-profit sectors.

## Declaration of competing interest

The authors declare that they have no known competing financial interests or personal relationships that could have appeared to influence the work reported in this paper.
