# Supplementary material for: Risk of infective endocarditis in patients with mitral valve prolapse: Systematic review with meta-analysis
Source: Heliyon. 2024 Nov 4;10(21):e39893. doi: 10.1016/j.heliyon.2024.e39893 (PMC11582404; doi:10.1016/j.heliyon.2024.e39893)
Supplement: Multimedia component 1 [file mmc1.pdf]

Supplementary material

Risk of Infective Endocarditis in patients with Mitral Valve Prolapse: Systematic review with meta-analysis

Table of contents

Supplementary material 1 - Databases and search strategies.. 3
Supplementary material 2 – Main characteristics of the included case-control studies. 4
Supplementary material 3 - Main characteristics of the included cohort studies........... 5
Supplementary material 4 – Risk of Bias assessment 6
Supplementary material 5 – Forest plot for NOS subgroups 7
Supplementary material 6 – Forest plot for study design subgroups............................. 8
Supplementary material 7 – Sensitivity analysis 9
Supplementary material 8 - Publication bias 9

Supplementary material 1 - Databases and search strategies

EBM Reviews - **Cochrane Central Register of Controlled Trials January 2022**, Ovid MEDLINE(R) and Epub Ahead of Print, In-Process, In-Data-Review & Other Non-Indexed Citations, Daily and Versions(R) 1946 to February 14, 2022

#	Searches
1	exp mitral valve prolapse/
2	(mitral adj4 prolapse).af.
3	(mitral adj4 floppy).af.
4	mitral AJD4 myxomatous.af.
5	1 or 2 or 3 or 4
6	endocarditis.af.
7	exp endocarditis/
8	6 or 7
9	5 and 8

Web of Science Core Collection

Mitral (all fields) and prolapse (all fields) and endocarditis

SciELO Citation Index

Mitral (topic) and prolapse (topic) and endocarditis (topic)

Supplementary material 2 – Main characteristics of the included case-control studies

Author, year	Cases demographic data	Cases characteristics	IE criteria	Confounding factors for IE	Control selection	MVP criteria
**Clemens, 1982**	Yale – New Haven Hospital (November 1976 – November 1980)	32 men, 41 women<br>Mean age: 47 ± 18 years	▪ Pathological documentation<br>▪ Clinical criteria (heart murmur + 2 positive blood cultures + peripheral stigmata / laboratory evidence)	Exclusion of patients with HD antecedents<br>No exclusion of patients with previous dental work<br>Posterior analysis of IDUs	Adult inpatients undergoing echocardiography	Systolic leaflet displacement ≥2mm
**Danchin, 1989**	CHRU de Nancy – Brabois (January 1981 – January 1986)	33 men, 14 women<br>Mean age: 55.0 years	▪ Pathological documentation<br>▪ Fever + 2 major clinical criteria<br>▪ Fever + 1 major + 3 minor clinical criteria<br>(major criteria: 2 positive blood cultures, new heart murmur, vegetation on echocardiography; minor criteria: arterial embolism, immunologic disorders, splenomegaly, regurgitation murmur, clinical signs of vasculitis, CHF, pre-existing HD)	No exclusion of patients with HD antecedents, patients with previous dental work or IDUs	Subjects < 60 years undergoing routine echo + patients admitted for surgery of the limb	Abnormal arc of one / both mitral leaflets above the level of the mitral valve annulus into the left atrium
**Devereux, 1986**	New York Hospital – Cornell Medical Center (November 1978 – December 1982)	49 men, 18 women<br>Mean age: 38 ± 14 years	▪ Positive blood cultures<br>(one patient only had clinical criteria – fever, arthralgias, rash, anemia, hematuria, elevated ESR)	No exclusion of patients with previous dental work<br>Posterior analysis of patients with HD antecedents or IDUs	Randomly selected subjects + Patients undergoing echocardiography + Unselected subjects with MVP	Systolic leaflet displacement ≥2mm
**Hickey, 1985**	Prince Henry and Prince of Wales Hospital Group (January 1976 – January 1984)	32 men (51 ± 15 years)<br>24 women (45 ± 21 years)	▪ Heart murmur + two positive blood cultures<br>▪ Pathological documentation + evidence of infection (pyrexia, embolism, immune complexes)	Exclusion of patients with HD antecedents<br>No exclusion of patients with previous dental work<br>Posterior analysis of IDUs	Inpatients undergoing echocardiography	Leaflet displacement ≥2mm or pansystolic posterior displacement ≥ 3mm with mitral leaflet echoes
**Strom, 1998**	Delaware Valley Case-Control Network (August 1988 – November 1990)	Sex: N/A<br>Mean age: 59.1 ± 17.1 years	▪ Clinical judgment per review of medical records	No exclusion of patients with HD antecedents, patients with previous dental work or IDUs	Community control using the Waksberg random-digit dialing method	N/A (interview)

CHF – congestive heart failure; ESR – Erythrocyte Sedimentation Rate; HD – heart disease; IDUs – intravenous drug users; IE – infective endocarditis; MVP – mitral valve prolapse

Supplementary material 3 - Main characteristics of the included cohort studies

Author, year	Demographic data	Characteristics of MVP cohort	MVP criteria	Characteristics of IE cases	IE criteria	Confounding factors for IE
**Katan, 2016**	Olmsted County residents with MVP (January 1989 – December 1998)	331 men, 565 women<br>Mean age: 53 ± 21 years<br>Follow-up period: 11 ± 5 years	Systolic leaflet displacement ≥2mm	3 men, 5 women<br>Mean age: 62 ± 22 years	Modified Duke criteria	No exclusion of patients with HD antecedents, patients with previous dental work or IDUs

HD – heart disease; IDUs – intravenous drug users; IE – infective endocarditis; MVP – mitral valve prolapse

Supplementary material 4 – Risk of Bias assessment

Newcastle – Ottawa Scale	Selection				Comparability	Exposure / Outcome			Total
	1	2	3	4	1	1	2	3	
**Clemens, 1982**	0	1	0	1	1	1	1	1	6
**Danchin, 1989**	1	1	1	1	1	1	1	1	8
**Devereux, 1986**	1	1	1	1	1	1	1	1	8
**Katan, 2016**	1	1	1	1	2	0	1	1	8
**Hickey, 1985**	1	1	0	1	1	1	1	1	7
**Strom, 1998**	0	1	1	1	2	0	1	0	6

NEWCASTLE - OTTAWA QUALITY ASSESSMENT SCALE: CASE CONTROL STUDIES

Selection:

1) Case definition adequate: 1 point if IE diagnosis confirmed with echocardiographic or pathologic findings; 0 points if only based on medical records

2) Representativeness of the cases: 1 point if representative case series; 0 if potential for selection bias

3) Selection of controls: 1 point if community controls; 0 points if hospital controls or no description

4) Definition of controls: 1 point if no history of IE

Comparability: 1 point if study adjusted for age and sex; 1 more point if study adjusted for any other important risk factors

Exposure:

1) Ascertainment of exposure: 1 point if validation of MVP with echocardiography based on current criteria; 0 points if medical record only or self report

2) Same method of ascertainment for cases and controls: 1 point if yes

3) Non-response rate: 1 point if same rate for both groups

NEWCASTLE - OTTAWA QUALITY ASSESSMENT SCALE: COHORT STUDIES

Selection:

1) Representativeness of exposed cohort: 1 point if study population truly or somewhat representative of a community/population-based study; 0 if study population was sampled from a special population or not described

2) Selection of non-exposed cohort: 1 point if drawn from the same community as the exposed cohort; 0 if different community or not described

3) Ascertainment of exposure: 1 point if validation of MVP with echocardiography based on current criteria

4) Demonstration that outcome was not present at start of study: 1 point if IE diagnosis followed the MVP diagnosis

Comparability: 1 point if study adjusted for age and sex; 1 more point if study adjusted for any other important risk factors

Outcome:

1) Assessment of outcome: 1 point if IE diagnosis confirmed by echocardiographic or pathologic findings; 0 if only based on medical

Supplementary material 5 – Forest plot for NOS subgroups

Considering the risk of bias, an analysis based on the NOS scale was conducted. When comparing studies with NOS ≤ 7 (OR 8.09, 95% CI 3.98, 19.91; I2 = 37.18%) and NOS > 7 (OR 7.12, 95% CI 3.98, 12.73; I2 = 0%), there was a small difference on the values obtained, as seen below.

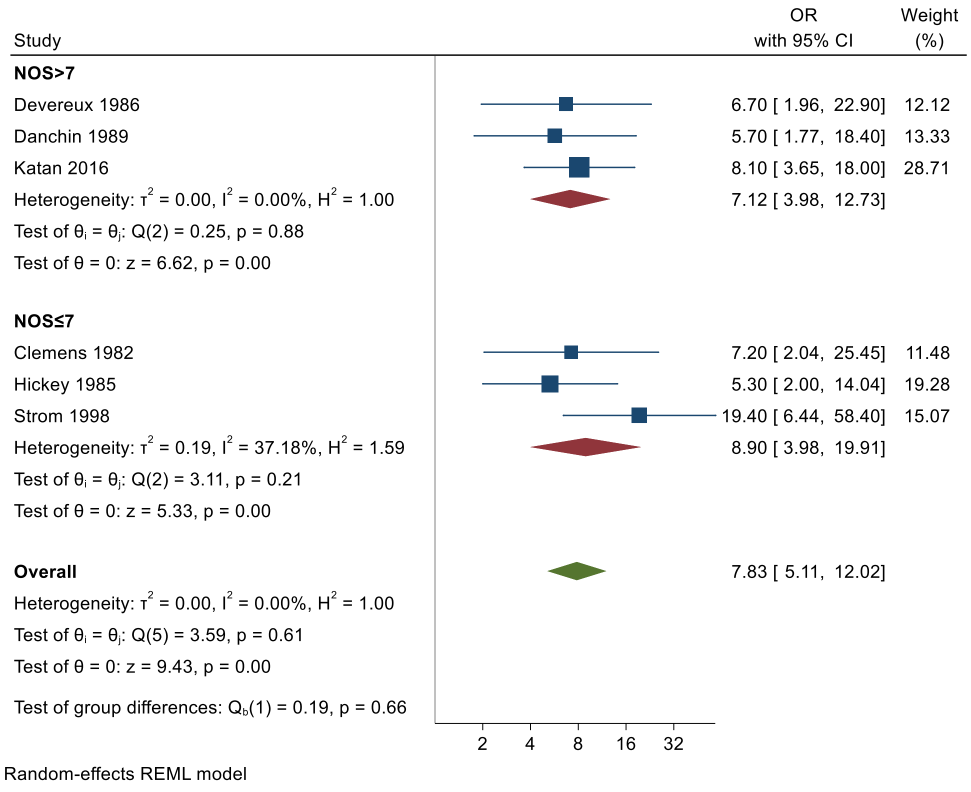

Forest plot with odds ratio and 95% CI for the studies with a NOS > 7 and for the studies with a NOS ≤ 7.

Supplementary material 6 – Forest plot for study design subgroups

An analysis was conducted to see possible differences regarding the study design. Both the case control (OR 7.73, 95% CI 4.66, 12.83; I2 = 0%) and the cohort (OR 8.10, 95% CI 3.65, 18.00; I2 = 0%) subgroups obtained a similar OR, as can be seen below.

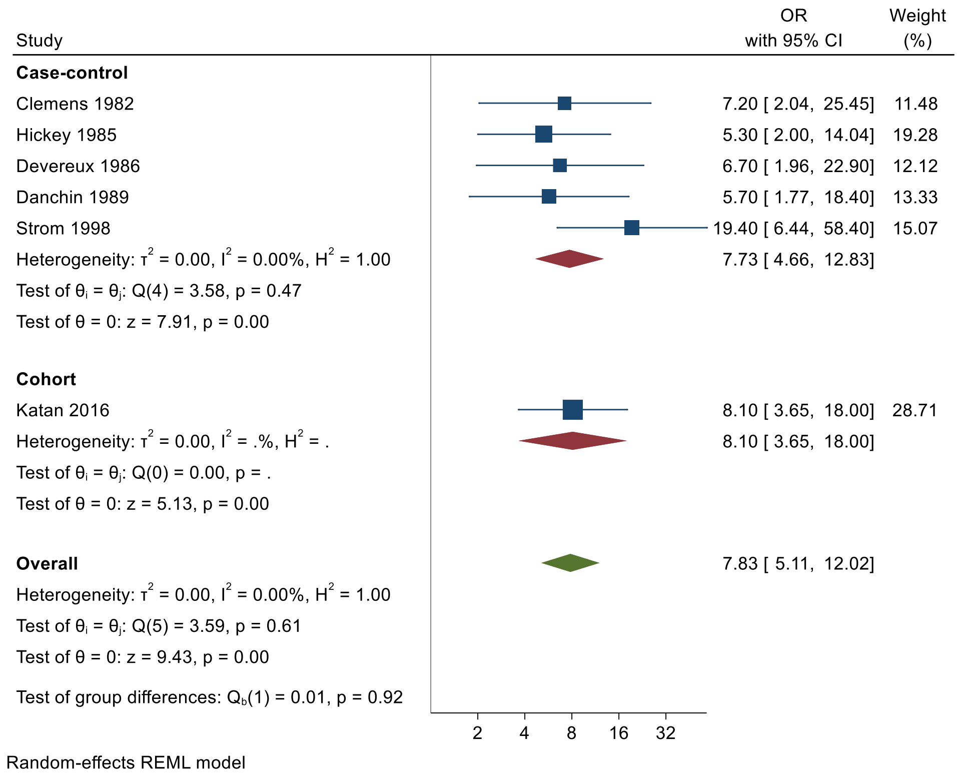

Forest plot with odds ratio and 95% CI for the case-control studies and the cohort study

Supplementary material 7 – Sensitivity analysis

Sensitivity analysis revealed that no studies were causing significant changes in the obtained OR. Regarding the studies with a higher risk of bias, analysis excluding Clemens et. al, revealed an OR 7.92 (95% CI 5.03, 12.48), similar to the values obtained in the overall analysis; however, exclusion of Strom et. al revealed an OR 6.67 (95% CI 4.19, 10.61), slightly inferior to the value obtained.

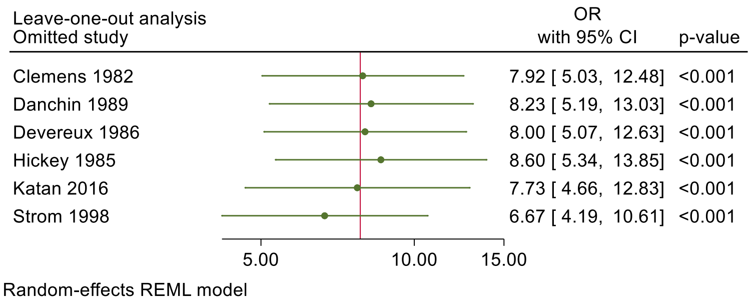

Forest plot with odds ratios and 95% CI obtained in the sensitivity analysis (leave-one-out analysis)

Supplementary material 8 - Publication bias

The funnel plot and the Egger test results (p=0.99) do not support publication bias.

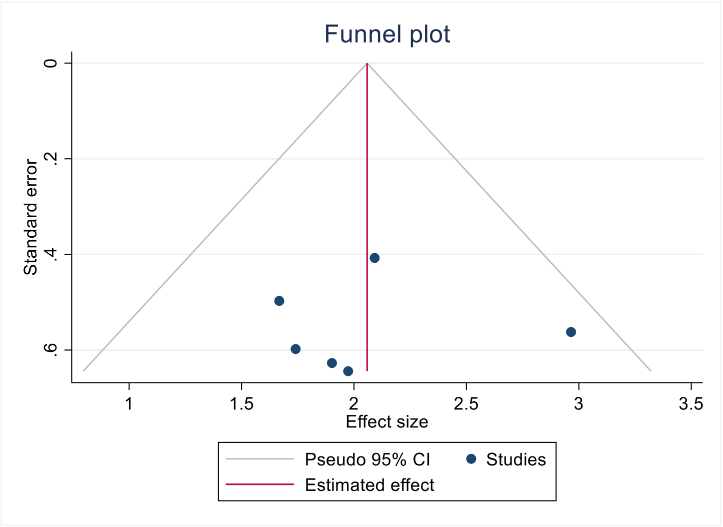

Funnel plot of the publication bias analysis
